# Therapeutic Goals of One-on-One Viniyoga: A Qualitative Study of Practitioner Perspectives and Case Applications

**DOI:** 10.3390/healthcare13192527

**Published:** 2025-10-06

**Authors:** Jennifer Vasquez, Michele Quintin Quill, Chase Bossart

**Affiliations:** 1 School of Social Work, Texas State University, San Marcos, TX 78666, USA; 2 Independent Researcher, Austin, TX 78745, USA; info@shifthappenstherapy.com; 3 Independent Researcher, Friday Harbor, WA 98250, USA; chase@yogawell.com

**Keywords:** Viniyoga, yoga therapy, mind–body interventions, integrative health, complementary health

## Abstract

Background/Objectives: Viniyoga is a person-centered approach to yoga that emphasizes individualized adaptation of breath, movement, and meditative practices to support health and well-being. This qualitative study investigates the therapeutic goals of one-to-one Viniyoga from the perspective of experienced therapists. Methods: Fourteen certified Viniyoga practitioners participated in in-depth interviews, which were analyzed using Interpretative Phenomenological Analysis (IPA). This study details how Viniyoga therapists define therapeutic Viniyoga. Results: The findings identify three core therapeutic goals that guide Viniyoga therapy: restoring balance, cultivating self-regulation, and guiding transformation. Two case studies are presented to illustrate the application of these goals in clinical contexts. Conclusions: Qualitative information provided by the interviewed Viniyoga therapists supports the positive role of individualized Viniyoga therapy in contributing to sustainable healing and supporting clients’ return to balance, self-regulation, and personal transformation. The Viniyoga therapeutic model is applicable across diverse populations and in a variety of integrative and complementary healthcare settings.

## 1. Introduction

Therapeutic Viniyoga is a dynamic approach to yoga founded on the teachings of T. Krishnamacharya and T.K.V. Desikachar that combines individualized practices of yogic movement, breath, sound, and meditative practices to increase well-being [1]. It emphasizes the importance of adapting yoga practices to meet the unique needs, conditions, and goals of each practitioner. While the broader field of yoga therapy has garnered increased academic attention, there remains limited empirical research specifically exploring how Viniyoga therapists conceptualize and apply therapeutic goals in one-on-one settings.

This study sought to identify and describe the primary therapeutic goals of Viniyoga, utilizing in-depth interviews with fourteen experienced Viniyoga therapists. In addition, this study defines therapeutic Viniyoga and includes two case studies to illustrate how these goals are implemented in Viniyoga therapy. This study aims to define the goals of Viniyoga therapy and increase insight into the lived experience and practical application of these goals. Thematic findings were developed using Interpretative Phenomenological Analysis (IPA), with a focus on practitioner experiences. These accounts offer detailed examples of client cases of healing through Viniyoga therapy, application of breath, movement, and the relationship between therapist and practitioner. By clarifying the goals of therapeutic Viniyoga, this research expands the literature on individualized yoga therapy and offers practical implications for therapists working with diverse populations and in a variety of clinical contexts.

### 1.1. What Is Therapeutic Viniyoga?

Viniyoga, a Sanskrit term meaning “application” or “appropriate use,” was adopted by T.K.V. Desikachar to describe the teachings of his father, T. Krishnamacharya [2]. These teachings, which are rooted in classical Indian philosophical systems and traditional yogic techniques, focus on an individualized application of yoga tools to holistically integrate a person’s unique needs of body, breath, mind, and spirit. The aim is to adapt the yogic philosophy and techniques to support the individual needs of each practitioner [2,3].

A core tenet of Viniyoga is its adaptability. Viniyoga practices are designed to accommodate an individual’s capabilities and to adjust as therapeutic goals develop over time [2]. Some of the central tools Viniyoga therapy utilizes include conscious movement, breathing techniques, directed attention, sound, and meditation. Unlike many modern yoga systems, Viniyoga emphasizes breath as the central axis of movement and incorporates both dynamic repetition and static holds in postures [1].

Therapeutic Viniyoga is an experiential and relational approach that incorporates somatic practices tailored to each client. When developing a therapeutic yoga practice, the Viniyoga therapist considers the client’s goals, lifestyle, time availability, physical and emotional condition, and familiarity with yogic practices [1]. Using this information, the therapist is able to create a purposeful, dynamic practice that promotes healing and transformation from within [4].

### 1.2. Distinguishing Viniyoga from Other Yoga Therapies

There are several different yoga therapies besides Viniyoga therapy. Perhaps the best known is Hatha yoga. A frequent understanding of Hatha Yoga is that it represents the union of opposites. As a practice of action, it engages both body and mind, calling for steady effort and discipline. Other well-known yoga styles, such as Kundalini yoga and Iyengar yoga, are considered different styles of Hatha yoga [5]. The question that arises is how to determine which yoga therapy style is most effective in meeting the varied needs of patients.

Several meta-analyses have considered the efficacy of different types of yoga therapy. While various studies have demonstrated benefits of Hatha yoga poses, a meta-analysis also noted that certain poses can cause harm to participants based on their individual physiological needs, necessitating the need for specific yogic guidance [5]. Another meta-analysis noted that a consistent finding of yoga therapy studies is that utilizing more components of yoga was more effective than using less, especially when combining breathing and meditation practices [6]. Finally, a meta-analysis of 306 randomized controlled studies found that while most studies concluded that yoga was beneficial regardless of style, 100% of the Viniyoga studies endorsed positive outcomes. The authors noted the low number of Viniyoga studies (N = 4), underscoring a need for more in depth exploration [7]. When considering these factors, Viniyoga stood out as a style that incorporates the same benefits of other yoga branches, such as poses, breathing, and meditation while offering a more individualized, flexible approach.

### 1.3. Understanding Therapeutic Viniyoga Goals

In broad terms, the goals of therapeutic Viniyoga are to apply individualized yogic practices to restore a person’s systemic balance, increase self-regulation skills, and guide lasting personal transformation. These aims are achieved through breath-centered, individualized tools that are responsive to a person’s unique constitution, condition, and context [2,8]. Thus, Viniyoga is a dynamic, interactive therapeutic process that relies on the input of both practitioner and client.

Through these individualized Viniyoga therapeutic tools, clients increase their inner awareness of their body’s systems and gain experiential understanding of how to better self-regulate. Both increased self-regulation skills and the resulting autonomic stability are essential factors in decreasing stress and emotional reactivity as well as improving cognitive functioning [9,10]. As well, individuals benefit from the specificity of Viniyoga therapy. Because Viniyoga therapy teaches repeatable practices that are individually tailored to each client’s goals and needs rather than generalized yogic sequences, clients develop a deeper connection to their own internal experience. The lived experience attuning to one’s system and creating positive change through Viniyoga practices increases a client’s self-efficacy for managing challenges [11].

Over time, practicing Viniyoga therapy can create a positive feedback loop for sustained personal change. As the client’s needs change, so do the Viniyoga therapy strategies employed. This allows the positive growth achievable through Viniyoga practice to keep up with the client’s changing needs. Simultaneously, the client becomes more knowledgeable about their systems and how to facilitate positive growth, which grants them greater agency and a heightened sense of self-efficacy. This enables the client to take lasting control of their healing process and successfully integrate it into daily living [12]. Therapeutic Viniyoga ultimately transcends symptom management to nurture a more aware, empowered, and resilient way of being.

### 1.4. Prior Research: Achieving an Optimal State

Modern Viniyoga therapists define the ‘optimal state’ as a condition classified by steady breath, heightened sensory interoception, emotional balance, and focused concentration [13]. This definition originates from classical Sanskrit texts such as Patañjali’s Yoga Sūtra, Haṭha Pradīpikā, the Bhagavad Gītā, and the Taittirīya Upaniṣad. These texts outline the characteristics of optimal human functioning across physical, energetic, emotional, mental, and spiritual dimensions. In Yoga Sūtra 1.2, Patañjali defines yoga as directing the mind in a chosen direction (citta-vṛtti nirodhaḥ), leading to clarity, stability, and integration [14]. Achieving an optimal state serves as a therapeutic goal within Vinyoga therapy, guiding both assessments and personalized interventions. These traditional markers are supported through modern research, such as research into neurobiological mechanisms like the enhancement of vagal tone and modulation of the default mode network [15,16]. Characterizing the optimal state using neural mechanisms can increase its identification beyond traditional yoga application into interdisciplinary fields such as neuroscience and psychology.

Neurophysiological and psychophysiological studies show that meditation and regulated breath practices, as emphasized in both the Haṭha Yoga Pradīpikā and modern Viniyoga, improve autonomic regulation, heart rate variability, stress resilience, and emotional regulation [17,18]. Furthermore, psychological balance and emotional resilience are supported by Viniyoga through the utilization of breath and movement as self-regulatory and distress tolerance skills. This is particularly relevant in the context of trauma-sensitive care, where body-based interventions have been studied to effectively decrease physiological arousal and increase emotional regulation and balance [19].

Such findings provide empirical grounding for the optimal state as both an ancient philosophical ideal and also as measurable psychological and physiological states. Viniyoga therapy employs the pañcamaya model to evaluate the practitioner’s current condition across five dimensions: body, breath, mind, personality, and spirit and provide appropriate interventions accordingly [20]. Viewed through the lens of both classic yogic philosophy and modern scientific evidence, Viniyoga therapy has the capability of creating deep personal transformation and supporting more balanced and unified psychophysiological states. Clients are not passive recipients of treatment but active participants in their healing. Therapists work collaboratively with clients to develop and refine home practices, fostering self-efficacy and sustainability over time. In this way, Viniyoga serves not only as a therapeutic intervention but also as a lifelong tool for health promotion and personal transformation.

### 1.5. Prior Research: Personal Transformation

While traditional yoga instruction and physical therapy practices standardize their practices, therapeutic Viniyoga concentrates on individualized practice, based on the principle that each person’s path to optimal well-being is unique. Thus, personal transformation is a core aim and desired outcome in therapeutic Viniyoga. Transformation encompasses not only physical recovery but also changes in emotional, mental, energetic, and spiritual dimensions. Practitioners facilitate these multidimensional changes by adapting practices to each individual’s physical and psychological context, guiding them toward meaningful and sustainable growth.

The Viniyoga transformational process is rooted in the concept of parinama, or change, which is a key principle in yoga philosophy. Therapeutic Viniyoga helps individuals create deeper self-awareness and stability using breath-centered movement, personalized sequences, and focused meditation. As practitioners and clients co-create practices aimed at self-regulation, insight, and empowerment, transformation becomes both a process and an outcome. This orientation toward inner change distinguishes Viniyoga therapy from approaches that focus solely on symptom reduction or functional improvement, positioning it instead as a holistic modality that addresses the full spectrum of human experience.

A growing body of qualitative and mixed-methods research highlights personal transformation as a central outcome of therapeutic Viniyoga. A qualitative study contrasting Viniyoga to the Feldenkrais method described how Viniyoga’s emphasis on the therapeutic relationship and individual application of techniques led to significant relief of lower back pain. Key findings include the importance of empowering self-observation, the role of deliberately engineering experiential processes, the importance of therapist-client trust, and the integral role of state change in facilitating healing [21]. These findings support personal transformation in therapeutic Viniyoga as a deliberate, not incidental, outcome facilitated through an individually structured experiential process.

Other quantitative and clinical studies further support the transformative qualities of Viniyoga therapy. In a randomized controlled 12-week trial conducted in a workplace setting, a Viniyoga Stress Reduction Program boasted an outcome of a 33% reduction in perceived stress, significantly outperforming both the mindfulness-based stress reduction and control groups [22]. A clinical study that examined individually tailored Viniyoga practices, which focused on gradual and adaptive for reducing chronic insomnia, concluded there were meaningful improvements in participants’ measures of anxiety, fatigue, and overall sleep quality [23]. These empirical findings align with Viniyoga’s philosophical underpinnings, Viniyoga meaning “appropriate application”, and support the idea that transformation is achieved not through prescriptive models but through ongoing adaptation, breath awareness, and inner observation [14,24].

### 1.6. Prior Research: Increased Self-Regulation

Therapeutic Viniyoga increases self-regulatory capacities by practicing mindfulness, attuned emotional awareness, and self-compassion. Increases in these traits are all linked to improvements in well-being and reduction in psychopathology. Research that utilized yoga immersion scales demonstrated that participants with high engagement in yoga show significantly better psychological well-being, positive self-dialog, and mindfulness compared to controls [25]. Lower levels of depression and anxiety correlated with this immersion provide evidence that sustained yogic practice such as Viniyoga can help create deep integration of mind and emotional states [26].

Research studies of pranayama and breath-centered practices, a key component of Viniyoga therapy, have also provided support for positive influence on autonomic regulation and stress modulation. Various studies have explored how yogic breathing techniques can reduce anxiety, enhance sustained attention, and promote emotional balance [19,26]. Both trauma-sensitive and therapeutic Viniyoga therapy focus on using breath-centered movement to moderate arousal and safely support connecting to vulnerabilities [14,27]. Personal transformation is facilitated through repeated, tailored sequencing which allows practitioners to safely reconnect with bodily sensations and emotional integration. This also facilitates integration between body and mind.

Broader studies of yoga therapy, including trauma-sensitive yoga, reinforce the evidence of Viniyoga’s capacity to build emotional resilience. Trauma-sensitive yoga has demonstrated utility in helping survivors of psychological trauma develop a greater sense of mind–body connection, emotional regulation, and ownership over their internal experience [28,29]. Viniyoga therapy studies have shown its effectiveness in influencing both controlled and autonomic breath functions [29,30]. Viniyoga therapy fosters this awareness through personalized sequencing, breathwork, and safe connection to internal states, with a similar goal of fostering long term healing.

## 2. Materials and Methods

### 2.1. Study Design

This qualitative study used Interpretative Phenomenological Analysis (IPA) to explore how experienced Viniyoga therapists conceptualize and apply Viniyoga as a therapeutic modality in one-to-one contexts. IPA is grounded in phenomenology, hermeneutics, and idiography [31] and is particularly appropriate for examining how individuals make sense of their experiences. This study aimed to understand how Viniyoga is defined and applied therapeutically by trained practitioners, identify the central goals of therapeutic Viniyoga, and examine the philosophical and practical concepts guiding its application. Given IPA’s orientation, the focus was on achieving deep, contextual insight into the lived experiences of a carefully selected group of expert practitioners, rather than generalizing to a wider population.

### 2.2. Participant Recruitment

A purposive sampling strategy was used to recruit 14 experienced Viniyoga therapists trained in the tradition of Sri T. Krishnamacharya and T.K.V. Desikachar. Participants were selected to ensure philosophical alignment with Viniyoga’s individualized adaptive approach to yoga therapy. Inclusion criteria were completion of formal training in Viniyoga therapy from recognized schools including American Viniyoga Institute, Yoga Well Institute and Krishnamacharya Yoga Mandiram with a minimum of 3 years of Viniyoga therapy experience, and de-identified case examples they are willing to provide from their yoga therapy practice. Twelve of the fourteen participants held IAYT certification (C-IAYT); the remaining two were highly experienced practitioners without formal certification. Participants were recruited through direct outreach via email lists and professional networks, as well as snowball sampling to ensure a diverse representation of experience levels, geographic locations, and training backgrounds.

Sample size in IPA studies is typically small to allow for detailed analysis [31]. Saturation is typically reached within the first 12 interviews when participants are relatively homogenous [32]. In this study, saturation was defined as the point at which no new themes or categories emerged from the data. By the 12th interview, consistent and repetitive patterns had formed across participants. The final two interviews added richness and depth but did not introduce new conceptual categories. The resulting sample size of 14 was deemed sufficient to balance depth with thematic completeness, while ensuring qualitative validity.

Findings from these interviews also formed the basis for three previously published articles that explored central aspects of Viniyoga therapy: state change [27], health and healing [33], and the therapeutic application of one-to-one Viniyoga [12].

### 2.3. Data Collection

Following approval from the Institutional Review Board (IRB), all participants provided written informed consent prior to participation. Interviews were conducted via Zoom, lasted between 45 and 90 min, and were audio-recorded and transcribed verbatim. A semi-structed interview guide (Appendix E) was developed in consultation with two senior Viniyoga therapist educators to ensure content validity. The interview guide included open-ended questions exploring participants’ reflections on the goals, principles, and definition of therapeutic Viniyoga, tools and techniques used, views on health and healing, roles of the therapist-client relationship and illustrative case examples. This open-ended structure supported depth and flexibility while maintaining consistency across interviews.

### 2.4. Sample

Participants ranged in age from 31 to 80 years and identified as either female (n = 8) or male (n = 6). Thirteen participants identified as Caucasian and one as Asian/Pacific Islander. Academic backgrounds were diverse, including two doctorates, seven master’s degrees, and five bachelor’s degrees. Geographically, participants were based primarily in the United States, with one participant from Europe.

Therapists’ years of yoga practice ranged from 11 to over 40 years, and their professional yoga therapy experience ranged from 3 to over 26 years. Nine therapists practiced yoga therapy full-time and five part-time. They worked with clients experiencing conditions such as chronic pain, anxiety, cancer, trauma, and stress-related disorders, in both in-person and online settings. These characteristics are reflected in the case study presentations, where anonymized demographic information is included for context and comparability.

### 2.5. Data Analysis

**Figure A1:**
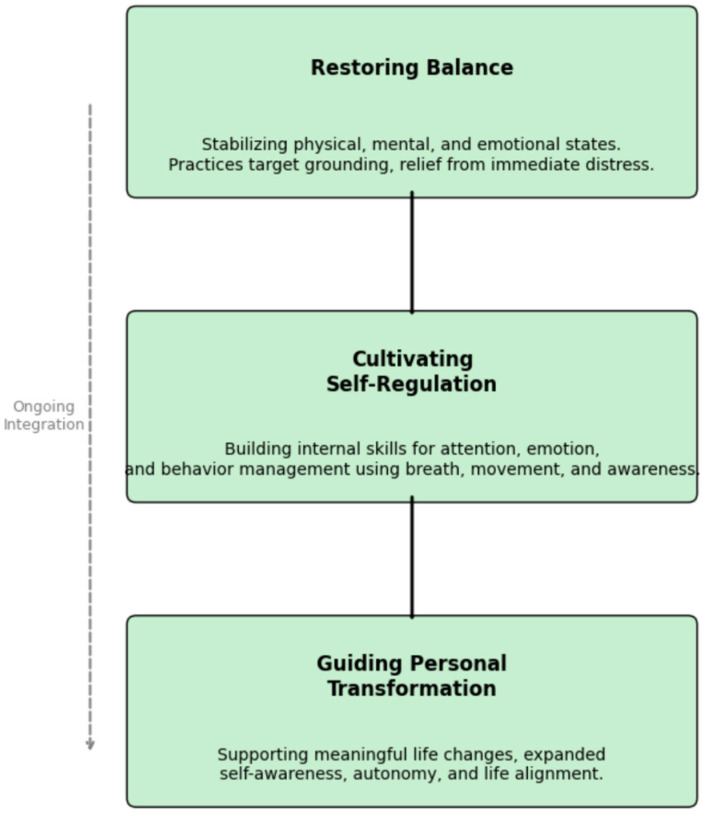
Conceptual Framework of the Three Therapeutic goals in Viniyoga Therapy.

Data were analyzed using a seven-stage IPA process: 1. Reading and Re-Reading: Immersion in each transcript to develop a holistic understanding. 2. Initial Noting: Detailed margin notes captured descriptive, linguistic, and conceptual observations. 3. Emergent Theme Development: Initial notes were synthesized into concise, interpretive themes. 4. Case-Level Theme Mapping: Emergent themes were grouped into overarching themes within each transcript. 5. Cross-Case Analysis: Recurring themes across participants were compared to build a thematic framework. 6. Thematic Consolidation: Overarching themes were refined and consolidated into a conceptual map (Figure A1). 7. Narrative Construction: Findings were woven into a coherent narrative supported by participant quotes [31].

Initial coding and theme development were conducted by the lead researcher, a Viniyoga-trained and IAYT-certified yoga therapist, who employed bracketing to consciously set aside personal experiences and assumptions during the data collection and analysis process. A secondary analyst independently reviewed a subset of transcripts and the initial thematic framework. Regular meetings were held to compare interpretations, discuss discrepancies, and achieve analytic consensus. Divergent perspectives were addressed through collaborative dialog and revisiting the raw data. Manual coding was supported using NVivo 14 qualitative software to organize data, track code frequencies, and manage evolving theme structures.

To further enhance objectivity, a co-investigator with no background in Viniyoga contributed an outsider’s viewpoint in order to balance the interpretation of the findings. The combination of these measures aimed to ensure that the analysis accurately reflected the participants’ perspectives while also upholding methodological rigor.

### 2.6. Ethical Considerations

Participants were informed of their rights, including the right to withdraw at any time. No compensation was offered. All data were anonymized and stored securely. Ethical approval was granted by the Lead Author’s Institutional Review Board, Protocol 9143.

### 2.7. Thematic Relationships

During analysis, three primary therapeutic goals were identified: Restoring Balance, Cultivating Self-Regulation, and Guiding Transformation. While interrelated, each theme captures a distinct dimension of the therapeutic process. For example, restoring balance involves stabilizing physiological and psychological systems, whereas cultivating self-regulation focuses on skill-building and client agency. Guiding transformation reflects existential and identity-level changes. Conceptual boundaries were clarified in the results section, and overlaps are acknowledged where appropriate.

To aid clarity and reader comprehension, Figure A1 provides a visual model showing how the three therapeutic goals intersect and interact. This conceptual map reflects the cyclical and layered nature of therapeutic Viniyoga, where balance facilitates self-regulation, which in turn supports deeper transformation.

### 2.8. Transparency and Reproducibility

To strengthen transparency, the interview guide is provided (Appendix F), direct quotes are included, and a synthesis of theme prevalence is presented (Table A1, Table A2, Table A3 and Table A4). A conceptual diagram (Figure A1) illustrates the interrelationship between the themes, clarifying areas of overlap while highlighting their distinct contributions to the therapeutic process. Anonymized case studies are used to contextualize findings

## 3. Results

This study aimed to (1) explore how practicing Viniyoga therapists define therapeutic Viniyoga and (2) identify the primary goals associated with its application. Through Interpretive Phenomenological Analysis of in-depth interviews with 14 experienced therapists, a nuanced definition of therapeutic Viniyoga emerged, along with key themes that characterize its therapeutic goals.

### 3.1. Understanding How Practicing Viniyoga Therapists Define Therapeutic Viniyoga

In exploring the core principles of therapeutic Viniyoga, participants consistently emphasized its individualized, adaptive, and relationship-centered nature. This form of yoga therapy is not a standardized set of practices, but rather a dynamic, responsive process in which the tools of yoga, such as breathwork, movement, meditation, sound, and attention, are individually incorporated to meet the specific needs, capabilities, and goals of each participant. Study participants described therapeutic Viniyoga as being practiced one-on-one. The aim is to encourage healing, support balance, and guide meaningful changes in physical, mental, and emotional states. As asserted by Participant 7, “The beauty of Viniyoga lies in its adaptability. Each session feels like a new journey, crafted uniquely for where I am at that moment”, capturing the essence of personalized transformation. This section presents participant perspectives that collectively define therapeutic Viniyoga as a personalized, holistic modality grounded in the intentional and skilled application of yoga’s full range of tools.

Practitioners widely understand the transformational nature of therapeutic Viniyoga as being connected to its personal application of yoga tools. “Therapeutic Viniyoga applies the tools of yoga specifically to the individual’s needs and their capacity in order to direct their entire system towards a state change or a state of balance,” Participant 12 explained. Other participants agreed, with Participant 14 describing it as “a customized practice to meet the current needs of the individual or group” (Participant 14) and that it involves “helping someone with a health condition or problem using the approach and tools of yoga in the way that is personalized for the individual” (Participant 11).

Core components of Viniyoga include breath, movement, attention, sound, and meditation, all of which are selected and adapted based on the person’s goals and context. Participant 4 noted, “I would say that it is using and applying yoga tools, especially breathing and attention, to help affect change in someone’s body and system.” These tools are designed not only to relieve symptoms such as pain or anxiety but also to address the systemic causes of imbalance. For instance, Participant 4 further explained, “If someone has pain, physical pain, then there might be tools like movement, breathing, meditation focuses that can help change the way the system is making decisions around the pain area so that that experience of pain is reduced or the sort of angst associated with being in pain is reduced.”

Therapeutic Viniyoga is also defined by its delivery format, which emphasizes individualized, one-on-one instruction. “The definition is essentially the application,” stated Participant 9. “If it is yoga therapy in the Viniyoga lineage, it is applying the body of knowledge of yoga and the tools of yoga in a therapeutic way whereby the focus is on the health issues is of the client. Moreover, the yoga is adapted to that.” Participant 13 similarly emphasized the relational aspect of the work: “Appropriate application of yoga given by a certified Yoga Therapist working one-on-one with a student.”

Ultimately, therapeutic Viniyoga is a holistic, evolving practice rooted in the belief that healing occurs through personalized, intentional engagement with yoga’s full spectrum of tools. As Participant 5 summarized, “Viniyoga uses the techniques and principles of yoga to create change in the practitioner’s life and state and hopefully promote healing,” adding that “all of those [tools] can be used, but are individually tailored is and that changes over time.”

### 3.2. Goals of Therapeutic Viniyoga

Through interpretative analysis of participant interviews, three interconnected goals of therapeutic Viniyoga emerged: (1) restoring balance, (2) enhancing self-regulation, and (3) supporting transformation. These goals reflect how Viniyoga therapists view the therapeutic process and the intended outcomes for clients. While individualized in application, these goals offer a shared framework for understanding how Viniyoga supports healing.

### 3.3. Restoring Balance

Participants described the central aim of therapeutic Viniyoga as supporting individuals in restoring balance across all levels of the human system, using the Pañcamaya model. Viniyoga facilitates meaningful change by aligning the whole system toward balance and sustainable health. Participants emphasized that the overarching goal of therapeutic Viniyoga is to support individuals in returning to a state of balance and optimal functioning. Participant 7 noted that Viniyoga is a method to help individuals align with their essential self, by removing obstacles across all layers of the Pañcamaya model. Participant 12 emphasized the importance of the individualized approach in tailoring techniques to address each individual’s needs and capacity in order to restore balance. This was echoed by Participant 4, who discussed the importance of initial assessment in identifying imbalances. This forms the basis for selecting specific techniques, such as breathwork, movement, or relaxation practices. According to Participant 10, this process involves starting with a simple practice and gradually adding additional dimensions as needed. Participant 5 explained, “You are always working to bring someone back into balance and then also expand their capacity.”

Physical and physiological outcomes were cited as markers of success. For example, Participant 3 described a client successfully reducing blood pressure and resting heart rate to normal ranges after continued Viniyoga therapy practice. Participant 7 reports a client regained knee function and reduced pain after surgery while practicing Viniyoga therapy. Participant 14 reports experiencing that Viniyoga therapy both alleviated physical pain, discomfort and reduced mobility, but also increased emotional balance as demonstrated by increased mental focus and emotional peace.

Ultimately, balance in therapeutic Viniyoga is achieved by utilizing a client-centered approach that applies the tools and principles of yoga on an individual, flexible basis (Participants 5 and 11). Clients are empowered to increase both their awareness and participation in their own healing practice (Participant 6). This allows the practitioner to develop coping skills that increase both symptom management and mind–body awareness (Participant 3). Because individuals often have secondary psychological and physiological responses to distress (e.g., feelings of despair regarding illness/psychological distress, tensing in response to pain), increasing self-efficacy and bodily attunement is a key component of restoring balance and increasing symptom reduction.

### 3.4. Cultivating Self-Regulation

A key theme described by study participants is how regular participation in Viniyoga therapy increased self-regulation by helping clients recognize and shift unhelpful psychological patterns by regulating internal states and increasing self-compassion and feelings of agency. Clients felt more empowered to navigate the challenges they faced. This emerged through a process of personal insight, emotional processing, and state transformation. These changes often unfolded alongside physical healing but extended far beyond symptom management, encompassing shifts in perception, self-relationship, and behavioral patterns.

By helping individuals better understand and change their relationship with their conditions, clients were able to benefit from increased clarity, improved mood, and more adaptive coping skills. As Participant 3 explained, “we are working with the relationship that they have to their condition and the symptoms of the condition,” especially in cases involving new diagnoses or chronic illness. This shift from identifying with suffering to creating space around it was essential for psychological healing. “When you remove the obstacle,” Participant 7 noted, “the result would be the person feeling better”, not just physically, but emotionally and mentally as well.

This process often led to greater emotional spaciousness. For instance, Participant 7 described a client whose personality and emotional response needed to shift concerning knee pain: “She needed some space emotionally,” and over time developed the ability to navigate her healing with less control and more acceptance. Similarly, Participant 13 shared how another client became more capable in caregiving while simultaneously releasing the need to control every outcome, reflecting a deepening emotional resilience.

Healing was not seen as a linear process, but rather a broad and individualized journey. Participant 4 emphasized that yoga therapy supports people “with an extensive range of interests or concerns,” including deeper explorations of human nature and spirituality. One client, over two years, joined a walking group, built social connections, and experienced a renewed sense of faith, all of which contributed to a feeling of being “supported by something larger, both God and organization.”

Therapeutic Viniyoga facilitates these transformations through state change and shifts in how individuals experience their thoughts, emotions, and body. Participant 10 explained that healing the mind involves moving from states of agitation (rajas) and inertia (tamas) to a state of clarity and harmony (sattva): “That is important, certainly for healing the mind. It is critical.” Participants reported being able to observe these changes in their clients “You can see a kind of ease in that person in their body, ease with themselves,” said Participant 8.

Another critical dimension of developing self-regulation skills is the client’s ability to discern what worsened or supported symptoms. “She began to become more aware of behaviors at home that were problematic,” shared Participant 14. Others noted that clients began to “feel more relaxed,” “take rest where they did not before,” and “stabilize emotionally” (Participant 12). This evolution often resulted in increased self-compassion. As one client expressed, “It is okay to feel this way, and I am allowed to have these feelings” (Participant 12).

Processing unresolved traumas and emotional wounds enhanced self-regulation skills for many practitioners. Participant 4 shared, “Healing can also include emotional wounds that shape our ideas and our behavior,” Effectively processing these past experiences helped practitioners orient to the present rather than reliving the past. The capacity to reflect on and integrate these experiences effectively led to shifting perspective from self-criticism to self-love, or from despair to joy, even in the face of chronic or terminal illness. Thus, balance is achievable even while participants still face adversity or unresolvable conditions.

Emotional regulation is more than resolving immediate symptoms; it is also about building resilience and systemic change across the different symptoms, as described by the Pañcamaya model. As Participant 14 explains, “Everything in the human system is linked. If someone has a state change the entire system and experience will change as a result.” With sustained, persistent practice, practitioners often experience sustained improvements in mood regulation, self-empowerment, and overall quality of life. These overarching improvements demonstrate deep emotional healing.

### 3.5. Guiding Transformation

Participants interviewed affirmed the capacity for Viniyoga therapy to guide long-term physical healing along with psychological and emotional transformation. Over time, the Viniyoga practices that restore balance and cultivate self-regulation can result in a deepening of self-relationship and a profound shift in internal awareness. This awareness can lead to shifts that go beyond symptom relief to the development of new patterns of self-engagement characterized by compassionate self-engagement and renewed authenticity and resilience to external stressors. Sustained Viniyoga therapy practice can foster non-linear healing that transforms participants’ quality of life, spiritual development, and sense of identity. One practitioner reflected, “Over time, clients usually notice that they are relating to the world differently, and their overall experience of life has changed, and usually improved” (Participant 14). Another explained, “The process of therapeutic Viniyoga is about creating a new system… a different way of experiencing oneself,” emphasizing that clients “get a chance every day in their practice to have an experience of something different… in the direction of peace and ease and comfort” (Participant 12).

Even in the absence of physical healing, Viniyoga therapy clients can significantly reconceptualize their relationship to their illness or redefine their suffering. Therapeutic Viniyoga can provide a more functional framework for accepting the unchangeable and responding effectively to internal dysregulation. One participant described a client with cancer who, after years of Viniyoga practice, said, “I am doing the things that are important to me,” having “intentionally established relationships” and structured her life around what mattered most (Participant 4). Another practitioner observed that through regular reflection and practice, “She was able to know what was happening and use her tools to calm her down. She is studying yoga therapy now. It became a big thing in her life” (Participant 12).

For some, transformation encompasses changes in established daily routines and a commitment to pursuing meaningful activities. Spiritual shifts were also significant. Participant 12 noted a client “questioning her identity and her relationship to God,” which led to writing poetry and exploring creative expression. Others described healing as a broader existential journey: “Healing is a lifelong quest,” one participant explained, “not some sort of linear thing. It is about being in relationship with oneself, putting our attention on ourselves, being interested in and willing to care for ourselves” (Participant 12). This idea was echoed by Participant 9, who stated, “Healing is about the person’s relationship to the experiences they are having and have had. It is about their perception and behavior concerning the circumstances.”

Study participants described clients who experienced the deepest transformations as presenting as more grounded, emotionally regulated, and receptive to experience. As Participant 8 described, one client experienced “a more open, a more buoyant, a more stable, calm kind of feeling that they had their life restored to them where possibilities were open.” These sustained personal changes were often marked by greater trust in one’s inner capacity. “I think the most important thing for the Viniyoga therapist to do,” said Participant 5, “is to trust the person’s innate ability to heal to communicate that you have confidence in their ability.” Participants attributed these successful transformations to the power of the therapeutic relationship coupled with consistent personal practice.

## 4. Case Study One: Initial Goal Setting and Longitudinal Progression with Viniyoga Therapy

Because Viniyoga therapy is individualized, case studies offer valuable insight into the application of goal setting in practice. Participant 3 provided a detailed case, a woman in her early sixties, who presented with significantly elevated blood pressure, tachycardia, and thyroid dysfunction. She was also experiencing chronic stress associated with a demanding career in education and recent personal hardships. Although she had some experience with yoga, she had not maintained a consistent practice in recent years.

This case, beginning with the initial intake and continuing through several months of practice, illustrates how therapeutic goal setting is initially conceptualized and then reassessed. Observations from both the therapist and the client are included.

### 4.1. Initial Goal Setting

Goal setting began with a preliminary phone/email exchange with the client to identify the client’s reasons for seeking therapy and broad goals. With this contextual understanding, Participant 3 utilized the first session to conduct an in-depth interview with the client, not just on the presenting concerns, but also to develop a holistic view of the client’s overall functioning. The therapist noted that this intake process was structured around the pañcamaya model, a multidimensional framework that addresses the physical, energetic, mental, emotional, and spiritual layers of the individual. This approach helped build rapport and allowed space for the client to feel comfortable and heard.

Participant 3 identified the initial goals as restoring balance and increasing self-regulation through stress reduction. The first intervention was a gentle, breath-centered āsana to introduce mindful movement. Based on the client’s presentation, the therapist incorporated practices to help lengthen exhalation, starting with a supine position to promote relaxation and safety. In between sessions, the client was encouraged to track her blood pressure to provide objective feedback on her physiological state of arousal. Participant 3 was mindful of the client’s cardiovascular concerns and avoided introducing overly strenuous practices at the outset.

### 4.2. Longitudinal Progression

Participant 3 reports scheduling a set of four sessions to provide structure and continuity for the client. During these sessions, the practitioner and client refined the client’s home practice and focused on fostering a deeper awareness of physical and mental patterns. Participant 3 noted that the client began introducing other positive lifestyle changes, including discontinuing nightly alcohol usage and increasing physical exercise. Alongside Viniyoga therapy, the client also consulted healthcare providers to track her cardiovascular health. As the client’s practice developed, Participant 3 slowly introduced more physically engaging postures and deeper breath work, always tailored to her physiological tolerance and overall state. Balance postures were later incorporated to address self-identified deficits in proprioception. The therapist also discovered that the client tended to rush through printed instructions; therefore, guided audio recordings were offered for home use. The client expressed that the recordings were helpful in supporting her sustained attention during home practice.

Excerpts from the client’s diary are incorporated to include the client’s perceptions and insights. These reflections mirror the therapist’s observations while also providing a personal account of the internal shifts experienced by the client. “The guided breathing exercises have brought a newfound sense of calm and clarity to my daily routine,” the client wrote. Another entry highlights an emotional breakthrough: “I feel like I am becoming more attuned to my body and thoughts, and this awareness is empowering.” The client’s entries express how she experienced both increased emotional balance and self-regulation skills as she transformed over time.

The client also experienced significant physiological restoration as well. Over time, her blood pressure and resting heart rate normalized, requiring no further medical interventions. More importantly, the client reported subjective improvements in her well-being, including increased vitality, reduced distractibility, and a felt sense of returning to herself. Participant 3 noted that the client demonstrated “a visceral awareness” of the shift from sympathetic activation to parasympathetic regulation. By the end of the observed period, the client had become more consistent in applying breath awareness and Viniyoga techniques to regulate stress in real-time, particularly in high-pressure work situations. Participant 3 summarized the outcome by noting that the client was “showing up in her life in a different way”, illustrating the client’s improved emotional balance and self-regulation skills over time.

## 5. Case Study Two: Initial Goal Setting and Longitudinal Progression with Viniyoga Therapy

The second case study, a middle aged, professional female who presented with severe back pain and other undiagnosed dysfunctions, provided by Participant 8, offers insight into the transformative potential of therapeutic Viniyoga, particularly in clients who present with significant physical limitations and emotional distress. This case highlights the individualized and creative application of Viniyoga tools in promoting access to movement, breath, and self-awareness.

### 5.1. Initial Goal Setting

Participant 8 described the integral role of the practitioner performing their own Viniyoga practice before each client session in order to restore one’s own emotional balance. This helps “to be as perceptive and receptive as possible to take in all these different aspects” while holistically assessing the client. This emphasis on self-practice also highlights that achieving therapeutic goals of emotional balance and self-regulation is experiential and requires continuous practice.

In the first session, Participant 8 applied a multidimensional assessment drawing on the pañcamaya model, which included visual observation and experiential evaluation of the client’s body and breath. However, the therapist encountered a significant challenge in assessing the client who was unable to perform even the most basic movements requested during the physical assessment. Participant 8 recalled, “I could not get anywhere because every single thing they were asked to do, they could not do… Sitting, raising the arm. Standing up. There was nothing.”

With these limitations, the therapist was momentarily uncertain about how to proceed. By facing the situation with curiosity, the therapist was able to spontaneously identify a culturally informed intervention. Drawing upon Sanskrit terminology, Participant 8 introduced the word “Aham,” which denotes a profound, indestructible sense of self. The therapist gently chanted the word in a soft rhythm and invited the client to repeat it. The client engaged with the sound “Aham, Aham”, and for the first time in the session, began to move while vocalizing. According to the therapist, “Aham became the key… Movement started happening, and all kinds of other things fell into place.”

The therapist used this opening to guide the client through breath-coordinated movement. The primary goals for the initial practice were to introduce gentle joint mobility and encourage openness in both body and spirit. This experience marked a pivotal turning point in the client’s willingness and ability to engage physically, emotionally, and energetically.

### 5.2. Longitudinal Progression

Participant 8 noted that, in Viniyoga therapy, clients often experience substantial transformation within the first few sessions. For this reason, the therapist generally schedules a few initial sessions and then allows the client to take charge of scheduling follow-up sessions based on their perceived progress and level of independence. “There is an awful lot of growth that happens right at the beginning of change… then, once the client is ready, I pass the baton,” Participant 8 described.

Despite only returning for a few sessions, this client experienced notable physical and psychological transformation. She progressed from initially only being able to perform tadasana (mountain pose) to uttasana (standing forward bend), which Participant 8 noted was “a big step”. This advancement reflected not only improved mobility but also a shift in confidence, awareness, and self-expression.

Both therapist and client noticed increased emotional balance and greater emotional skills. The therapist noted that the client’s posture, breathing, and demeanor began to transform. With increased physical openness came emotional spaciousness: “If there is room in the body—room for movement not to be constricted, room for breathing not to be constricted, then there is also room for the person to open up into the fullness of themselves.” With Viniyoga therapy daily practice, the client reported relief of depression symptoms and increased vitality. The therapist also observed changes in the client’s affect and improved mood. “This person began to have space for them to be in the world, their uniqueness to be in the world. The definition of yoga is going where we have never been before.” This sentiment expresses the essence of the Viniyoga approach in supporting the client’s health and healing.

## 6. Discussion

At its core, therapeutic Viniyoga is a relational, experiential model that uses a dynamic, responsive process relying on the input of both student and teacher. As the work progresses over time, students are able to experience both acute and gradual changes in symptom amelioration. This results in greater harmony across different systems and an increased sense of agency and self-efficacy on the behalf of the student. This process clarifies how the interrelated goals of restoring balance, enhancing self-regulation, and supporting transformation operate during the Viniyoga therapeutic process.

The perceptions and case studies detailed by study participants contribute to a more robust understanding of the underlying goals and mechanisms of change through the application of Viniyoga therapy. The interrelated goals, as analyzed using Interpretive Phenomenological Analysis (IPA), demonstrate Viniyoga’s unique prioritization of tailored, experiential, and ongoing self-inquiry.

The qualitative findings of this study are supported by recent quantitative studies, such as Wolever [22] which reported a 33% reduction in perceived stress and increased sleep quality among participants of a Viniyoga Stress Reduction Program. Another study by Sherman demonstrated that a Viniyoga intervention produced significantly better reductions in chronic low back pain compared to traditional physical therapy [34]. Another study also demonstrated Viniyoga can positively influence breathing ease and oxygen saturation for cancer patients [30].

Previous studies have demonstrated that other yoga traditions can have positive effects for specific conditions, such as Iyengar yoga for treating lower back pain [35], or utilizing Hatha yoga to increase distress tolerance [36]. In fact, most yoga therapy studies endorsed positive effects regardless of the type of yoga used [7]. These studies illustrate that other forms of yoga therapy may also be effective interventions. However, it is difficult to directly compare Viniyoga therapy to other styles of yoga therapy because many studies do not define the style of yoga therapy utilized [7,37]. Nonetheless, two meta-analyses found that a defining factor to successful yoga therapy is persistent practice [6,38]. The importance of persistent practice was also emphasized by participants in our study.

Studying both the qualitative and quantitative studies on Viniyoga therapy explains how an individualized approach can nonetheless provide measurable positive outcomes for a wide variety of conditions and a diverse range of participants. While the individualized nature of Viniyoga therapy requires in-depth analysis and in-depth inquiry to truly understand, key components emerge to demonstrate that therapeutic Viniyoga has promising applications for returning to an optimal state, enhancing self-efficacy and facilitating sustained personal growth that engages both teacher and student in a shared, transformational study.

### 6.1. Implications

The adaptable nature of Viniyoga therapy has significant implications for its potential as a complementary, non-pharmaceutical intervention in contemporary healthcare systems. Clarifying the underlying goals of Viniyoga therapy serves as a framework for understanding its potential application to a range of psychological and physical conditions. Because Viniyoga therapy is a holistic model, this framework also explains the interconnectedness of balancing physical, emotional, and cognitive domains in order to foster an optimal state for the patient.

For example, consider the possibility of a primary care clinic integrating Viniyoga sessions alongside routine care. Patients experiencing stress and psychosomatic concerns are referred for Viniyoga therapy as well as traditional medical care. These sessions offer patients personalized breathwork and gentle movement. As a result, patients feel more holistically cared for and experience reduced stress and an increased sense of self-agency. Concrete examples like this could inspire key stakeholders to adopt Viniyoga as part of a holistic, person-centered approach. Previous research supports the advantages of adapting such an approach. Hapidou and Huang explored the convergence between yoga therapy and frameworks such as Cognitive Behavioral Therapy (CBT), Acceptance and Commitment Therapy (ACT), mindfulness meditation (MM), and the relaxation response (RR), noting shared features like increased self-awareness, emotional regulation, cognitive flexibility, and empowerment, all of which can lead to better patient outcomes [38]. Viniyoga’s unique contribution lies in its structured yet adaptable framework, which not only cultivates these outcomes but also supports a return to balance by meeting individuals exactly where they are and evolving alongside their personal development.

These findings highlight the potential for integrating Viniyoga into multidisciplinary care models. Previous research has highlighted the effectiveness of tailored yoga interventions in managing chronic musculoskeletal conditions. Studies, such as those by Sherman and associates, have demonstrated significant benefits of yoga-based therapy compared to conventional physical therapy [35]. From this foundation, the current study expands understanding by illustrating how tailored Viniyoga practice facilitates regulation of the autonomic nervous system, enhances emotional resilience, and long-term psychological growth. Qualitative insights from therapists and clients detail how Viniyoga therapy can transcend symptom relief to evolve into a transformational model of well-being over time. These findings highlight the profound potential for integrating Viniyoga therapy across existing physical and psychological treatments.

### 6.2. Limitations and Future Research

Limitations exist both in the scope of this practice and in the demographics of interviewed participants. While limited client quotes are included, only Viniyoga therapists, not clients, were interviewed by the researcher. This is a significant limitation on our research, as the findings are self-reported only through the perspective of Viniyoga therapists. Their perspectives may not align with their clients’ self-reported experiences. Future research could benefit from exploring the perspectives of Viniyoga therapy clients and comparing/contrasting perceived outcomes from both sides. Longitudinal studies of both therapists’ and clients’ perceptions, rather than the singular data point perspective utilized in this study, could provide more insight on the progression and sustainability of goal objectives over time.

In addition, all participants were trained within the Viniyoga therapy tradition. This creates a possibility for interpretive bias through the influence of shared theoretical perspectives and taught values. Moreover, clients who seek out Viniyoga therapy may already hold positive beliefs or expectations, which could contribute to their perceptions of positive outcomes. Ongoing research might consider the ways in which expectancy effects and pre-existing beliefs shape both engagement in and outcomes of Viniyoga therapy. Further research could also benefit from examining how Viniyoga therapy compares with other yoga therapy traditions or alternative holistic approaches.

Although the sample size (n = 14) was appropriate for the IPA methodology, larger sample sizes in future studies would enhance generalizability and enable subgroup analyses based on clinical conditions, demographics, or training backgrounds. Because there is very limited qualitative data on Viniyoga therapy, the researchers relied on self-citation of previously published peer-reviewed studies utilizing these same interviews, which introduces the possibility of bias.

## 7. Conclusions

Therapeutic Viniyoga offers a versatile and individualized approach that supports a wide range of physical, physiological, emotional, and psychological conditions. This study explores how Viniyoga therapists define Viniyoga therapy using the pañcamaya model to facilitate holistic growth. Several core therapeutic goals were identified, including increasing emotional balance, improving self-regulation, and supporting personal transformation. The adaptive, individualized nature of Viniyoga therapy explains how Viniyoga can be adapted to achieve these goals across a wide variety of psychological and physical conditions.

By clarifying these guiding principles, this study helps explain what differentiates Viniyoga therapy from other mind–body interventions. The findings explain the potential of Viniyoga therapy as a complementary, non-medicinal optional for increasing self-regulation, supporting health behavior change, and promoting long-term well-being. Healthcare providers could use these findings to both approach patients utilizing an individualized framework as well as coordinate with Viniyoga therapists to provide more holistic care. Future research that includes client perspectives and comparative analyses with other therapeutic modalities will be critical in further establishing Viniyoga’s role in integrative healthcare. Further empirical research is needed to identify which conditions Viniyoga therapy is most effective in treating as well as which aspects of Viniyoga therapy are most critical for positive outcomes.

## Data Availability

The data presented in this study are available on request from the corresponding author. The data are not publicly available to protect client confidentiality. Access to the data may be granted on a case-by-case basis, subject to appropriate ethical review and approval.

## Appendix B

**Table A1:** Prevalence of primary therapeutic goals among participants.

Theme	Number of Practitioners Mentioning	% of Sample (n = 14)
Restoring Balance	12	86%
Cultivating Self-Regulation	12	86%
Guiding Transformation	13	93%

## Appendix C

**Table A2:** Frequency Table of Participant Contributions to Theme 1: Restoring Balance.

Participant ID	Number of Quotes	Evidence of Contribution to Theme
Participant 1	4	Stillness, somatic system, ideal functioning, deep knowing
Participant 2	6	Breath coordination, diaphragm, symptom stabilization
Participant 3	5	Managing symptoms, blood pressure, chronic conditions
Participant 4	2	Health history, returning to balance
Participant 5	2	Balance and capacity, promoting healing
Participant 6	3	Diaphragmatic breathing, empowerment, multi-level support
Participant 7	4	Removing obstacles, Panchamaya, functional state
Participant 8	3	Harmonious state, first session goals
Participant 10	1	Layering practice over time
Participant 11	3	Stress reduction, life improvement, focused attention
Participant 12	3	State change, improved functioning
Participant 14	1	Symptom relief, mobility improvement

## Appendix D

**Table A3:** Frequency Table of Participant Contributions to Theme 2: Cultivating Self-Regulation.

Participant ID	Number of Quotes	Notes/Keywords from Contribution
Participant 1	9	Emotional processing, grief, boundaries, self-awareness, emotional recovery
Participant 2	11	Listening, trauma, addiction recovery, emotional healing
Participant 3	4	Relationship to condition, emotional regulation
Participant 4	14	Spirituality, support systems, trauma processing, perspective shift
Participant 5	1	Suffering alleviation, mental/emotional state change
Participant 7	5	Emotional space, mindset shift, stress recovery
Participant 8	2	Optimism, positive emotional state shift
Participant 9	3	Stress regulation, healing capacity
Participant 10	9	Client state observation, attunement, emotional transformation
Participant 12	5	Emotional agency, acceptance of illness, joy, perspective shift
Participant 13	3	Caregiving agency, improved mood, internal empowerment

## Appendix E

**Table A4:** Frequency Table of Participant Contributions to Theme 3: Guiding Transformation.

Participant ID	Number of Quotes	Key Concepts/Transformation Markers
Participant 1	30	Patterning, state change, system optimization, agency, decision-making, empowerment, confidence growth
Participant 2	4	Clarity, personal discipline, gradual transformation, practice commitment
Participant 3	4	Increased comfort, emotional presence, self-connection, consistency in practice
Participant 4	8	Grief integration, goal alignment, empowerment, faith revival, action on values
Participant 5	4	Self-healing belief, confidence in inner wisdom, therapist trust, expanded self-concept
Participant 6	1	Long-term reflection, non-coercive practice engagement
Participant 7	7	Shifting perspectives, self-kindness, spiritual growth, resistance to change, healing milestones
Participant 8	4	Renewed life outlook, improved functionality, stronger relationships, embodied healing
Participant 9	5	Refined practice, self-study, perceptual shift, deeper inner relationship, progressive healing journey
Participant 10	4	Freedom from limitation, adaptation, self-regulation, practice evolution
Participant 12	7	Identity shift, poetic expression, spiritual inquiry, creativity, wholeness concept
Participant 13	3	New life patterns, joyful living, deeper focus, value re-alignment
Participant 14	2	Changed worldview, improved relational engagement, holistic transformation

## Appendix F

Semi-structured Interview Guide.

Please answer the below questions from the perspective of your understanding and practice of therapeutic Viniyoga:

General Descriptions and Goals

1. Describe therapeutic Viniyoga, what is it?
2. What are the general goals of therapeutic Viniyoga? What are you intending to do/facilitate?
3. How does therapeutic Viniyoga work? Explain the mechanism(s) as you understand them.
4. Describe your process of therapeutic Viniyoga. What happens in an initial session with a client?
5. Describe your process of therapeutic Viniyoga, what happens over time?
6. What are some of the techniques you use most often in therapeutic Viniyoga.

Examples

7. Think about a specific client you helped.
  (a) What was this client’s presenting problem?
  (b) Describe your initial session–the support you perceived they needed, techniques you used, and plans to begin to address those goals.
  (c) Describe the progression of your work with that client. What were some of the milestones?
  (d) How did that client’s state change over the course of yoga therapy?
8. Think about a different specific client you helped.
  (a) What was this client’s presenting problem?
  (b) Describe your initial session–the support you perceived they needed, techniques you used, and plans to begin to address those goals.
  (c) Describe the progression of your work with that client. What were some of the milestones?
  (d) How did that client’s state change over the course of yoga therapy?

What is Health and Healing

9. As a Viniyoga therapist, how do you think about health and healing?
10. Are there stages you think about in healing? If so, what are they?
11. Describe the role, if any, of state change in Viniyoga’s approach to healing.
12. Describe state change, what is it?
13. Describe how you know that the client’s state has changed, what changes in the client? Does anything change in you?

Roles of Therapist and Client

14. What are the most important supports for the client in this process? Why?
15. What is most important for the Viniyoga Therapist to do? Why?
16. Is there anything important for the Viniyoga Therapist not to do? If so, what?
17. What is most important for the client to do? Why?
18. Is there anything important for the client not to do? If so, what?
19. What concepts do you consider most important in studying to apply Viniyoga therapeutically? And why is each one important?
20. Is there anything you would like to add?
